# Predictors of Nutritional Status, Depression, Internet Addiction, Facebook Addiction, and Tobacco Smoking Among Women With Eating Disorders in Spain

**DOI:** 10.3389/fpsyt.2021.735109

**Published:** 2021-11-26

**Authors:** Amira Mohammed Ali, Hiroaki Hori, Yoshiharu Kim, Hiroshi Kunugi

**Affiliations:** ^1^Department of Behavioral Medicine, National Institute of Mental Health, National Center of Neurology and Psychiatry, Tokyo, Japan; ^2^Department of Psychiatric Nursing and Mental Health, Faculty of Nursing, Alexandria University, Alexandria, Egypt; ^3^Department of Psychiatry, Teikyo University School of Medicine, Tokyo, Japan; ^4^Department of Mental Disorder Research, National Institute of Neuroscience, National Center of Neurology and Psychiatry, Tokyo, Japan

**Keywords:** body mass index/BMI, depression psychopathology, eating disorders, Facebook addiction/internet addiction, nutritional status, Spain/Spanish, tobacco smoking, women

## Abstract

Eating disorders (EDs) are a complex group of psychiatric conditions that involve dysfunctional eating patterns, nutritional alterations, and other comorbid psychopathologies. Some women with EDs may develop problematic internet use while they attempt to get information on dieting/weight control or get online support from people with similar problems. They may also drift toward tobacco smoking as a method to regulate their weight or to cope with their weight-related dysphoria. The occurrence of these conditions in EDs may prolong disease course and impede recovery. This study used structural equation modeling to investigate nutritional status (noted by body mass index, BMI), depression psychopathology, internet addiction (depicted by the Internet Addiction Test), Facebook addiction (depicted by the Bergen Facebook Addiction Scale), and smoking among 123 Spanish women diagnosed with EDs (mean age = 27.3 ± 10.6 years). History of hospitalization, marital status, age, and the level of education predicted BMI in certain ED groups. BMI did not predict depression, but it predicted internet addiction, Facebook addiction, and smoking in certain ED groups. Depression did not predict BMI, internet/Facebook addition, or smoking in any ED group. Some sociodemographic and clinical variables had indirect effects on depression, internet addiction, and Facebook addiction while age was the only variable expressing a direct effect on all outcome measures. Age, education, and history of prolonged treatment predicted smoking in certain ED patients. The findings signify that a considerable target for interventional strategies addressing nutritional and addictive problems in EDs would be women with high BMI, history of hospitalization, history of prolonged treatment, who are particularly young, single, and less educated. Replication studies in larger samples, which comprise various subtypes of EDs from both genders, are warranted to define the exact interaction among the addressed variables.

## Introduction

Eating disorders (EDs) are a complex group of psychiatric conditions entailing dysfunctional eating patterns that are associated with other mental and physical symptoms (1–3). Genome-wide investigations report higher polygenic scores for body mass index (BMI) among patients with anorexia nervosa (AN), bulimia nervosa (BN), and binge eating disorder (BED) (4). BMI is an indicator of nutritional status, which is obviously altered in EDs due to excessive dieting (e.g., underweight in patients with AN) or overweight/obesity due to excessive intake of calories in response to aversive environmental stimuli [e.g., in patients with BED and addictive eating disorders; (1, 4, 5)]. The latter involve less control over eating, frequent thinking about food, eating when not hungry, and even withdrawal-like symptoms when some foods (e.g., non-nutritive sugar and processed food) are not available (1). Frequent consumption of these foods is likely to increase the incidence of depression (6).

Depression is highly comorbid with EDs (7), and it occurs as a symptom of distress associated with discomfort about body shape and/or food consumption (2, 5, 8). Depressive symptoms in women with EDs also result from malnutrition/starvation as indicated by body weight, BMI, fat free mass index (FFMI), and serum level of beta-hydroxybutyric acid (2, 9, 10). Adipokines produced by fat tissue in obese individuals such as leptin stimulate the production of proinflammatory cytokines and free radicals resulting in a chronic general state of systemic inflammation and metabolic dysregulation (11, 12). As a result, brain injury (neuroinflammation and neurodegeneration) may develop, consequently triggering symptoms of depression (2, 12, 13). Weight restoration in women with AN is associated with a transient rise in depressive symptoms (9). In the meantime, large-scale data from UK Biobank denote an increased genetic susceptibility to high BMI in depressed individuals (14). Nonetheless, the interaction between BMI and dysphoric mood is complex entailing an interplay among several dietary, psychological, sociodemographic, and medication factors (2, 8, 13, 15–17).

While some EDs may have addictive eating demeanors (1), substance abuse is frequently comorbid in patients with EDs (7). Large-scale genome-wide studies report genetic association of EDs with substance abuse, depression, impulse control disorders, schizophrenia, and attention deficit hyperactivity disorder (4, 18, 19). University students who express symptoms of dysfunctional eating tend to exhibit excessive internet dependence (20). Patients with EDs may turn to digital interventions, which express efficacy comparable to that of face-to-face modalities (21), to keep anonymity. They may also refer to online patient groups to seek support, to learn acceptable ways for refusing food, and to share experiences conducive to weight loss, including some dangerous ways (22). Given the high prevalence of negative emotions (depression and anxiety) among patients with EDs (2), along with their genetic tendency toward the development of addictive disorders (4, 18, 19), individuals with EDs may shift toward internet addiction (20, 22)—repeated and uncontrolled use of the internet that causes deterioration of health, emotional negativity, poor impulse control, and maladjustment (23, 24). Depression, anxiety, and suicidal ideation are key correlates of problematic internet use (25). Thus, internet addiction may be a source of an extra emotional burden that furthers the deterioration of EDs by contributing to other addictive behaviors such as smoking. In fact, the literature associates internet addiction with tobacco smoking, heavy drinking, substance abuse, and sexual promiscuity in both genders, although at varying degrees (26–29). Negative emotions seem to be a core common effector in dysfunctional eating, internet addiction, and smoking (5, 17, 26, 30, 31).

**Figure 1:**
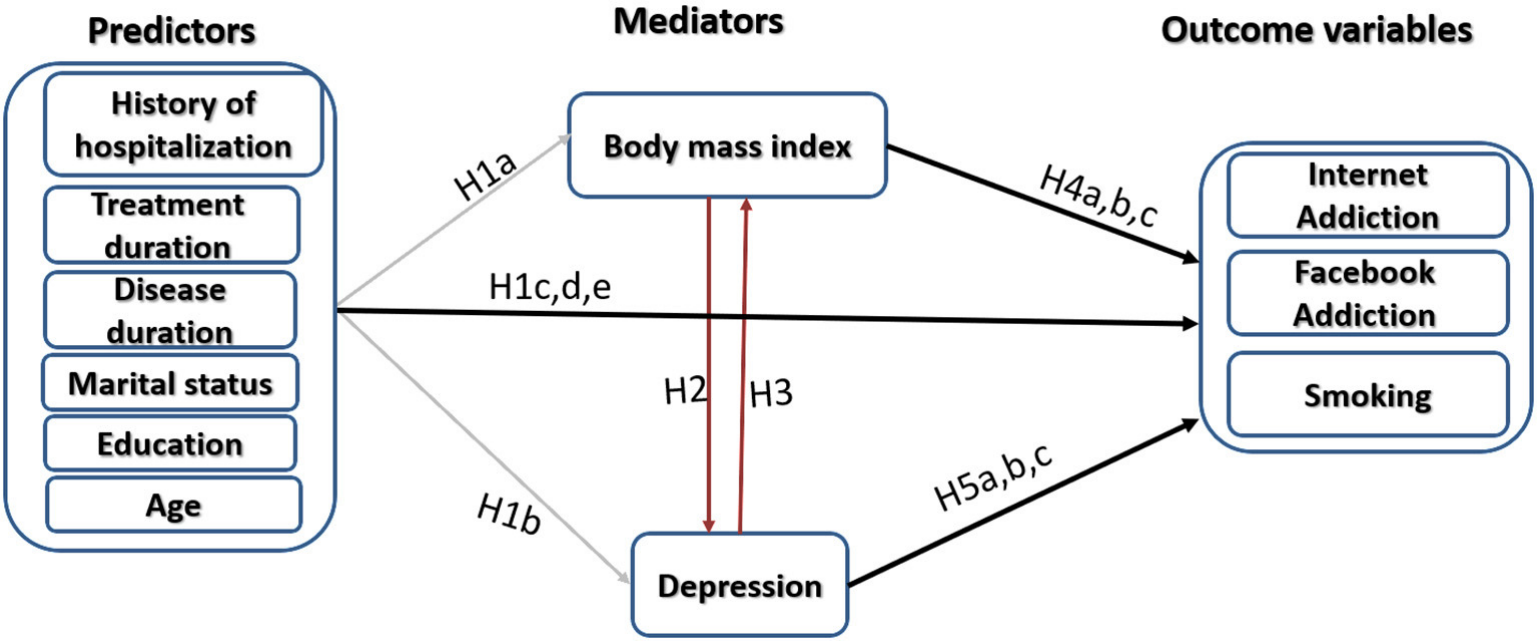
Schematic illustration of the model used to test hypotheses of the present study. Hypothesis 1a (H1a): Sociodemographic and clinical characteristics contribute to altered body mass index in patients with EDs. Hypothesis 1b (H1b): Sociodemographic and clinical characteristics contribute to depression in patients with EDs. Hypotheses 1c, d, e (H1c,d,e): Sociodemographic and clinical characteristics contribute to internet addiction, Facebook addiction, and smoking in patients with EDs. Hypothesis 2 (H2): Altered body mass index contributes to depression in patients with EDs. Hypothesis 3 (H3): Depression contributes to altered body mass index in patients with EDs. Hypotheses 4a, b, c (H4a,b,c): Altered body mass index contributes to internet addiction, Facebook addiction, and smoking in patients with EDs. Hypothesis 5a, b, c (H5a,b,c): Depression contributes to internet addiction, Facebook addiction, and smoking in patients with EDs.

Smoking rates are higher among women with EDs than in healthy control women (32). Smoking in EDs is associated with impulsive personality traits (32) and abuse of caffeine, alcohol, and marijuana (33). The literature spots a common association among eating behaviors, body weight, and smoking although the exact interaction between these first two variables and smoking is not well-defined (5, 34–36). While cigarette smoking may decrease appetite resulting in low BMI, some smokers tend to have high BMI and high waist circumference, indicating general and central obesity (5, 34). On the other hand, higher baseline BMI may trigger the initiation of smoking as an attempt to control or lose weight in some groups (35, 37). Analysis of data from UK Biobank and the Tobacco and Genetics (TAG) consortium (*N* = 372,791 and *N* = 74,035, respectively) shows that each SD increment in BMI increases the risk of being a smoker [odds ratio = 1.18, 95% CI 1.13–1.23, *p* < 0.001; (34)]. Smoking cessation is associated with weight gain probably due to increased caloric intake as a replacement of the smoking habit with food intake (34). Therefore, worries about gaining weight after smoking cessation may interfere with individuals' decision to quit smoking (35). The dynamics underlying the relationship between smoking and dysfunctional eating may be more complex than they seem to be. Imaging studies report abnormal activity of brain regions associated with self-referencing and emotional processing (e.g., medial prefrontal cortex and other brain regions) among smokers, AN and BN patients, and obese individuals who eat in response to aversive states (5, 30, 31, 38, 39). Psychopathological features closely linked to dysfunctional emotional processing are documented core correlates of internet addiction, smoking, binge drinking, substance abuse, problematic gambling, and maladaptive patterns of physical activity (26). A considerable attention should be paid to smoking among women since it is on the rise, especially in Europe (up to 38%) (40), and it is associated with increased incidence of metabolic disorders [e.g., diabetes mellitus; (41)], lung cancer (42), mortality (43, 44), and maternofetal complications when a smoker becomes pregnant (45). Based on the aforementioned background, the current study aims to identify the relationship between BMI and depression among women with EDs. It also hypothesizes that depression and BMI can predict internet addiction, Facebook addiction, and smoking in this patient group. These relationships are evaluated while taking into consideration the interaction of sociodemographic and clinical characteristics of the participants. Figure 1 summarizes the relationships addressed in the current study.

## Methods

### Study Design, Participants, and Procedure

This cross-sectional study comprises a sample of 124 women recruited consecutively from the General University Hospital of Ciudad Real in Spain during the period between February and November 2018. Participants were included if they were aged above 12 years, were treated for EDs either at the outpatient or inpatient level, and were willing to sign an informed consent. Having cognitive impairment or physical disabilities disqualified participation in the study (22). The analysis is based on a de-identified publicly accessible dataset (46). Therefore, ethical approval has not been obtained for the current study.

### Study Instruments

Data were collected through a self-administered questionnaire that consisted of three parts. Part 1 comprised a set of questions about participants' sociodemographic and clinical characteristics such as age, weight, height, ED diagnosis, disease duration, duration of being ever in treatment for EDs, history of hospitalization, psychiatric comorbidities, and smoking status.

The second part of the questionnaire comprised 20 items of the validated Spanish version of the Internet Addiction Test (IAT) (47). This scale was developed by Kimberly Young to measure problematic use of the internet. Items are rated on a 5-point Likert scale (1 = rarely, 2 = occasionally, 3 = frequently, 4 = often, and 5 = always) to depict the frequency of the symptoms. The total scores of the IAT range between 20 and 100; scores below 42 indicate normal use while higher scores indicate higher levels of internet dependence (24, 48, 49). The scale expresses sound psychometric properties including excellent internal consistency in the current sample (Cronbach's alpha = 0.99) (24).

The third part of the questionnaire comprised six items of the validated Spanish version of the Bergen Facebook Addiction Scale [BFAS; (50)]. Items of the scale identify problematic use of Facebook based on a score that ranges between 1 (very rarely) and 5 (very often). Problematic Facebook use can be detected at a total score of 12 or above based on a score of 3 on each of the six items of the BFAS (22). The internal consistency of the BFAS in the current study is excellent (Cronbach's alpha = 0.99).

### Statistical Analysis

Because one response was missing, it was removed from the dataset resulting in a sample size of 123. We have used the overall score of the IAT and the BFAS in the statistical analysis. Both variables were tested for normality of distribution by Shapiro–Wilk and Kolmogorov–Smirnov tests of skewness and kurtosis. Descriptive statistics of non-normally distributed variables are reported as median (MD) and interquartile range (IQR) while numbers and percentages are used to describe dichotomous and categorical variables such as having a diagnosis of depression and smoking. Spearman's rho test was used to examine correlations. We created two groups of EDs: AN and other EDs. This is because AN was the most common diagnosis in the sample, and suboptimal body weight and low BMI were eminent features in this group. Meanwhile, other EDs were less presented, and BMI indicated overweight and obesity in patients with BN and BED; it was close to overweight in patients with EDs not otherwise specified. Therefore, these three conditions were grouped together (other EDs) to make subgroup analysis more informative. Between-group differences in sociodemographic and clinical variables were compared using independent sample *t*-test and χ^2^-test, with bootstrap based on 1,000 random samples.

Normality testing revealed that the IAT and the BFAS were not normally distributed. Analyzing non-normal data derived from large samples by maximum likelihood (ML) and weighted least squares methods of estimation yields similar results—in terms of overall fit and the discrepancy between estimated parameter values and the true parameter values used to generate the data (51). Therefore, we fitted the hypothesized structural equation (SEM) model using ML with bootstrap involving the generation of 2,000 random samples. We tested if the model fits differently in the two EDs groups through multigroup analysis. A non-significant χ^2^ index was used to signify global model fit (24). Meanwhile, a good model fit was based on Comparative Fit Index (CFI) and Tucker–Lewis Index (TLI) equal to or above 0.95, and root mean square error of approximation (RMSEA) and standardized root-mean-square residual (SRMR) <0.06, although <0.08 was considered for an acceptable fit (13, 52).

Because ML model examining the predictors of smoking expressed poor fit, we created a Bayesian model to identify the distribution of the predictors of smoking. This is because smoking is a dichotomous variable (smoker, non-smoker). Bayesian modeling estimates the posterior distribution of parameters in question (predictors of smoking) as the mean of the distribution while uncertainties in these parameters are reported as credible intervals (CrI), which enclose 95% of the distribution of the potential predictors (44). A good model fit is judged by a posterior predictive *p*-value of around 0.5 and a symmetric 95% CrI centering close to zero (53). We created an initial model including depression, BMI, age, IAT, BFAS, marital status, education, years since diagnosis, years in treatment, and history of hospitalization as predictors. Because the posterior predictive *p*-value of this model was very low (0.00), indicating non-fit, most non-significant demographic and clinical variables were gradually eliminated until a good fit was obtained—at the point noting no further change in model fit, insignificant predictors (mainly BMI or depression) were retained. The analysis was conducted in SPSS and Amos version 24. Significance was considered at a probability of 0.05, two-tailed.

## Results

The current sample comprised 123 women with EDs; AN (*N* = 59, 48.0%), BN (*N* = 35, 28.5%), BED (*N* = 11, 8.9%), and eating disorders not otherwise specified (*N* = 18, 14.6%). The sociodemographic and clinical characteristics of the participants are reported in Tables 1, 2. For all the sociodemographic and clinical characteristics, there were no statistically significant differences between patients with AN and patients with other EDs except for BMI *t*_(73.0)_ = −6.77, *p* = 0.000 and history of hospitalization $\chi_{\left(1\right)}^{2}$ = 34.56, *p* = 0.000. Marital status and internet addiction showed a trend toward significance $\chi_{\left(1\right)}^{2}$ = 3.80, *p* = 0.051; and *t*_(110.1)_ = −1.94, *p* = 0.054.

**Table 1:** Sociodemographic and clinical characteristics of the participants in the samples.

**Participants' characteristics**	**Whole sample (*N* = 123)**	**AN (*N* = 59)**	**Other EDs (*N* = 64)**
	**No (%)**	**No (%)**	**No (%)**
Age mean (SD) in years	27.3 (10.6)	25.8 (11.3)	28.7 (9.8)
**Marital status** 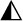			
Single	100 (81.3)	**52 (88.1)**	**48 (75.0)**
Married	22 (17.9)	**7 (11.9)**	**15 (23.4)**
**Education**			
Below high school	5 (4.1)	2 (3.4)	3 (4.7)
High school	78 (63.4)	41 (69.5)	37 (57.8)
University	40 (32.5)	16 (27.1)	24 (37.5)
**Ever hospitalized**			
Yes	63 (51.2)	**47 (79.7)**	**16 (25.0)**
No	60 (48.8)	**12 (20.3)**	**48 (75.0)**
BMI mean (SD)	22.2 (8.4)	**17.8 (2.6)**	**26.1 (9.7)**
Years since diagnosis MD (Q1–Q3)	8.0 (3.0–16.0)	7.0 (3.0–15.0)	9.0 (3.0–18.0)
Years of treatment MD (Q1–Q3)	6.0 (2.0–12.0)	6.0 (2.0–14.0)	6.0 (2.3–12.0)

*AN, anorexia nervosa; ED, eating disorder; 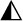, one observation is missing; BMI, body mass index; MD, median; Q1, first quartile; Q3, third quartile. Values in bold face are significantly different between groups*.

**Table 2:** Descriptive statistics of internet addiction, Facebook addiction, smoking, and depression in the samples.

**Key variables**	**Whole sample (*****N*** **=** **123)**		**AN (*****N*** **=** **59)**		**Other EDs (*****N*** **=** **64)**	
	**MD**	**Q1–Q3 (IQR)**	**MD**	**Q1–Q3 (IQR)**	**MD**	**Q1–Q3 (IQR)**
IAT	16	5–35 (30)	15	4–33 (29)	16.5	5–54 (49)
BFAS	33	23–60 (37)	34	24–50 (26)	32	23–70.8 (48)
Smoking no (%)	56	45.5%	23	39.0%	33	51.6%
Depression no (%)	36	29.3%	15	25.4%	21	32.8%

*AN, anorexia nervosa; ED, eating disorder; IAT, Internet Addiction Test; BFAS, Bergen Facebook Addiction Test; MD, median; Q1, first quartile; Q3, third quartile; IQR, interquartile range*.

**Figure 2:**
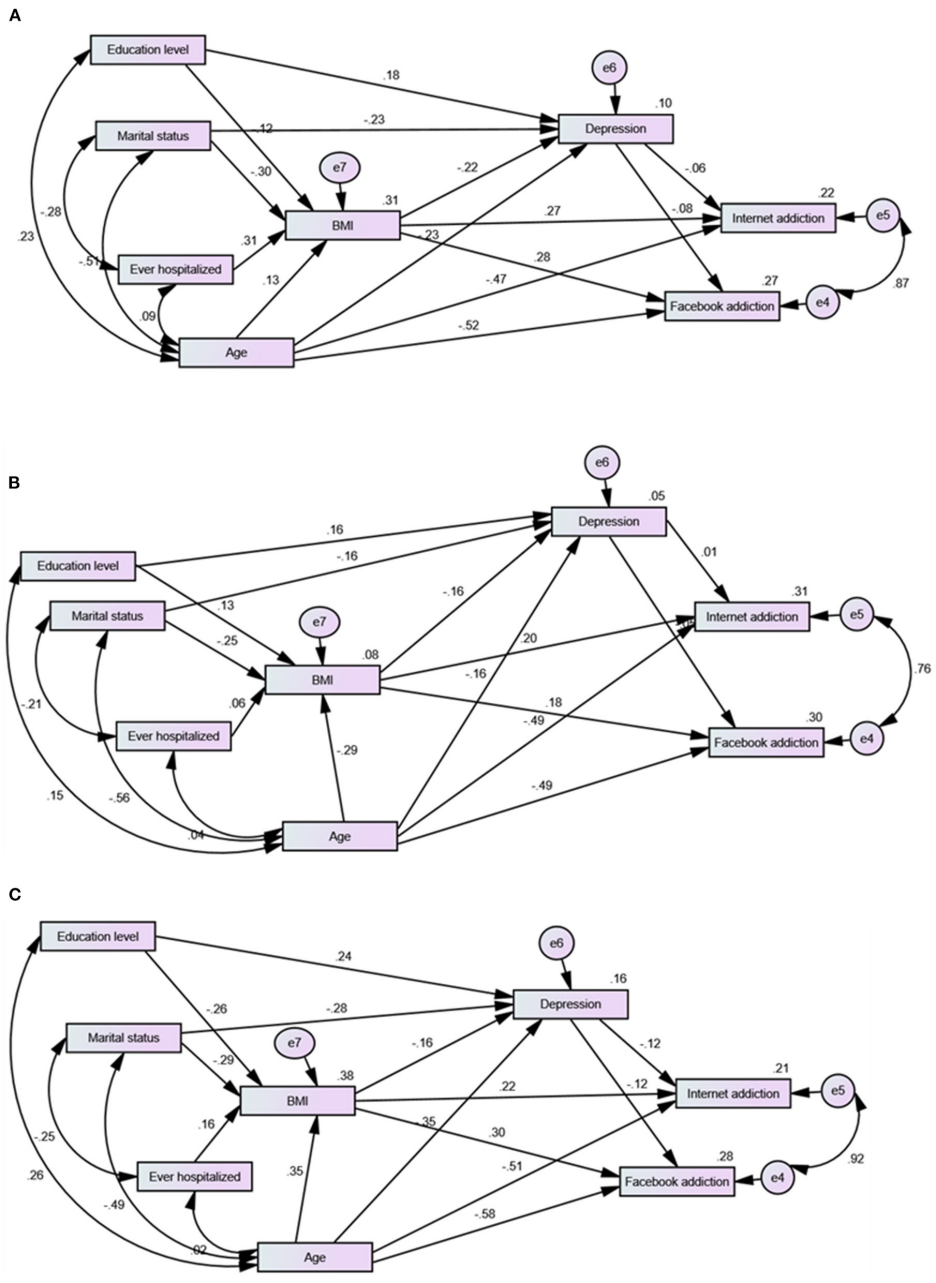
Direct effects from structural equation model predicting depression, internet addiction, and Facebook addiction among women with different eating disorders **(A)**, with anorexia nervosa **(B)**, and with other eating disorders **(C)**.

The non-significant χ^2^ index of overall model fit resulting from ML with bootstrap analysis shown in Table 3 indicates that the used models fitted the data on a global basis. Meanwhile, other absolute fit indices indicate good fit of the models tested in the samples. Figure 2 displays the direct effects of predictor and mediator variables on BMI, depression, internet addiction, and Facebook addiction while Table 4 shows the total effects, including indirect effects. For all patients, patients with AN, and patients with other EDs, in order, SEM models accounted for 31, 8, and 38% of the variances in BMI; 10, 5, and 16% of the variances in depression; 22, 31, and 21% of the variances in internet addiction; and 27, 30, and 28% of the variances in Facebook addiction (Figure 2).

**Table 3:** Fit indices of the tested basic model in the samples.

**Samples**	**χ^**2**^**	***df***	***p***	**CFI**	**TLI**	**RMSEA**	**SRMR**
Whole sample (*N* = 123)	9.570	9	0.386	0.998	0.995	0.023	0.0489
AN (*N* = 59)	12.437	9	0.190	0.970	0.908	0.081	0.0837
Other EDs (*N* = 64)	4.582	9	0.869	1.000	1.069	0.000	0.0414

*AN, anorexia nervosa; ED, eating disorder; χ^2^, chi-square; df , degrees of freedom; CFI, Comparative Fit Index; TLI, Tucker–Lewis Index; RMSEA, root mean square error of approximation; SRMR, standardized root mean residual*.

**Table 4:** Standardized regression weights of the total effects of predictors of internet addiction and Facebook addiction along with *p*-values and 95% confidence interval.

**Predictors**	**Samples**	**Outcome variables**											
		**Body mass index**			**Depression**			**Internet addiction**			**Facebook addiction**		
		**β**	***p***	**95% CI**	**β**	***p***	**95% CI**	**β**	***p***	**95% CI**	**β**	***p***	**95% CI**
Education	*N* = 123 *N* = 59 *N* = 64	−0.117 0.127 −0.263	0.194 0.392 **0.036**	−0.294 to 0.055 −0.153 to 0.401 −0.502 to −0.016	0.209 0.144 0.280	**0.022** 0.310 **0.041**	0.031 to 0.369 −0.113 to 0.331 0.016 to 0.502	−0.044 0.027 −0.092	0.131 0.385 **0.035**	−0.123 to 0.015 −0.039 to 0.143 −0.217 to −0.006	−0.050 0.015 −0.114	0.108 0.622 **0.022**	−0.133 to 0.010 −0.058 to 0.130 −0.254 to −0.015
Marital status	*N* = 123 *N* = 59 *N* = 64	−0.296 −0.255 −0.289	**0.040** 0.104 0.154	−0.525 to −0.010 −0.614 to 0.049 −0.571 to 0.096	−0.170 −0.121 −0.236	0.067 0.418 0.085	−0.353 to 0.017 −0.414 to 0.223 −0.498 to 0.048	−0.069 −0.053 −0.037	0.092 0.116 0.410	−0.162 to 0.013 −0.172 to 0.017 −0.175 to 0.075	−0.067 −0.039 −0.058	0.117 0.248 0.310	−0.160 to 0.018 −0.155 to 0.034 −0.188 to 0.069
Ever hospitalized	*N* = 123 *N* = 59 *N* = 64	0.311 0.058 0.155	**0.001** 0.505 0.143	0.167 to 0.448 −0.121 to 0.244 −0.050 to 0.345	−0.068 −0.009 −0.025	**0.022** 0.363 0.216	−0.163 to −0.007 −0.084 to 0.014 −0.145 to 0.010	0.087 0.012 0.037	**0.002** 0.374 0.116	0.029 to 0.173 −0.019 to 0.079 −0.008 to 0.127	0.091 0.011 0.050	**0.001** 0.351 0.120	0.033 to 0.180 −0.016 to 0.077 −0.011 to 0.146
Age	*N* = 123 *N* = 59 *N* = 64	0.135 −0.294 0.350	0.229 0.093 **0.019**	−0.082 to 0.347 −0.581 to 0.055 0.048 to 0.620	−0.259 −0.111 −0.406	**0.022** 0.520 **0.004**	−0.469 to −0.038 −0.492 to 0.256 −0.670 to −0.108	−0.422 −0.554 −0.384	**0.001** **0.002** **0.001**	−0.542 to −0.291 −0.669 to −0.397 −0.575 to −0.196	−0.461 −0.540 −0.425	**0.001** **0.001** **0.000**	−0.580 to −0.326 −0.677 to −0.362 −0.617 to −0.246
BMI	*N* = 123 *N* = 59 *N* = 64	–	–	–	−0.217 −0.156 −0.158	**0.027** 0.254 0.274	−0.445 to −0.022 −0.405 to 0.114 −0.515 to 0.124	0.279 0.202 0.241	**0.003** **0.037** 0.057	0.093 to 0.442 0.011 to 0.383 −0.009 to 0.469	0.294 0.185 0.322	**0.002** 0.095 **0.011**	0.114 to 0.460 −0.033 to 0.387 0.075 to 0.532
Depression	*N* = 123 *N* = 59 *N* = 64	–	–	–	–	–	–	−0.059 0.009 −0.117	0.474 0.913 0.308	−0.229 to 0.103 −0.0215 to 0.224 −0.344 to 0.102	−0.084 −0.051 −0.125	0.330 0.741 0.281	−0.250 to 0.083 −0.288 to 0.198 −0.348 to 0.096

*N = 123, whole sample; N = 59, anorexia nervosa sample; N = 64, other eating disorders sample; β, standardized total parameter estimates. Boldface values indicate significant findings*.

As shown in Table 4, BMI predicted depression, internet addiction, and Facebook addiction in the whole sample. Its effect on internet addiction and Facebook addiction was marginal in both ED groups. Age predicted depression in the whole sample as well as BMI and depression in women with other EDs. It was the only sociodemographic variable that had a direct significant effect on internet addiction and Facebook addiction in all the samples. Education predicted depression in all the participants as well as BMI, depression, internet addiction, and Facebook addiction in women with other EDs. Its effect on the last two was indirect—mediated by BMI. Marital status predicted BMI in the whole sample. Depression had no effect on BMI (path trimmed), internet addiction, and Facebook addiction in all the samples.

To examine if this basic model significantly varies between patients with AN and patients with other EDs, we ran multigroup analysis involving both groups. Multigroup analysis indicates overall agreement of the tested model across ED groups. However, constraining regression weights, covariances and residuals to equality between groups considerably reduced model fit (Supplementary Material). Some significant effects noted in the whole sample were insignificant in one or both groups due to several between-group variations in the shared group means of BMI, internet addiction, and Facebook addiction as well as regression weights of marital status and age in the paths predicting depression and BMI (Supplementary Material).

As for predictors of smoking in the participants, the tested Bayesian models fitted data properly in all the samples as noted by posterior predictive values close to 0.5. As shown in Table 5, age was the only significant sociodemographic predictor of smoking in the whole sample while age and education were significant predictors of smoking in patients with AN. BMI predicted smoking only in patients with AN. On the other hand, neither age nor BMI predicted smoking in patients with other EDs. Depression was not a significant predictor of smoking in all the samples (paths trimmed in the whole sample and other EDs group), and mediation analysis showed no indirect effects of age or BMI on smoking via depression in patients with AN (−0.001 ± 0.000, 95% CrI: −0.017 to 0.012 and −0.015 ± 0.000, 95% CrI: −0.095 to 0.042, respectively). Years in treatment and chronicity (years since diagnosis) predicted smoking in the whole sample and in patients with other EDs.

**Table 5:** Predictors of smoking among the samples indicated by posterior probabilities from Bayesian modeling.

**Predictors**	**Whole sample (*****N*** **=** **123)**				**AN (*****N*** **=** **59)**				**Other EDs (*****N*** **=** **64)**			
	**Mean**	**SD**	***p*▴**	**95% CrI**	**Mean**	**SD**	***p*▴**	**95% CrI**	**Mean**	**SD**	***p*▴**	**95% CrI**
Depression	–	–	–	–	0.325	0.308	0.46	−0.149 to 0.990	–	–	–	–
BMI	–	–	–	–	0.182	0.086	0.46	**0.024 to 0.361**	0.000	0.019	0.45	−0.036 to 0.037
Age	0.049	0.021	0.47	**0.008 to 0.092**	0.040	0.019	0.46	**0.003 to 0.079**	–	–	–	–
Level of education	0.204	0.123	0.47	−0.037 to 0.447	0.578	0.215	0.46	**0.164 to 1.007**	–	–	–	–
Years in treatment	0.855	0.309	0.47	**0.257 to 1.474**	–	–	–	–	0.258	0.091	0.45	**0.091 to 0.436**
Years since diagnosis	−0.120	0.046	0.47	–**0.210 to** –**0.032**	–	–	–	–	−0.179	0.072	0.45	–**0.328 to** –**0.048**

*AN, anorexia nervosa; ED, eating disorder; ▴, posterior predictive p; 95% CrI, 95% credible interval. Boldface values indicate significant finding*.

## Discussion

This study investigated the predictors of BMI and if BMI predicts depression among patients with EDs. It also examined the possible effects of BMI and depression on problematic use of the internet and Facebook as well as tobacco smoking, taking into account the effect of some of the confounding factors reported in the literature. Sociodemographic and clinical characteristics, not depression, predicted BMI. BMI was not associated with depression, but it was associated with internet addiction, Facebook addiction, and smoking in certain ED groups. Depression did not predict BMI, internet/Facebook addition, or smoking. Some sociodemographic and clinical factors had indirect effects on depression, internet addiction, and Facebook addiction while age was the only variable expressing a direct effect on internet addiction and Facebook addiction. Age, education, and few clinical characteristics predicted smoking in certain ED patients.

History of hospitalization and marital status predicted BMI in the whole sample while age and education predicted BMI in patients with other EDs. These findings are consistent with those of previous reports. In this regard, BMI in hospitalized patients with AN increased from admission to discharge, which was likely to be explained by lower admission BMI and longer inpatient stay (54). BMI ≥19 kg/m^2^ at hospital discharge is associated with five-fold higher BMI restoration in women with AN (55). A study investigating the clinical impact of marital status on ED symptomatology suggests that ED patients who live with a partner are significantly older and demonstrate significantly higher levels of purging behavior than non-cohabiting counterparts (56). Our study supports this report as indicated by a significant correlation between being ever hospitalized and marital status (*r* = −0.284, *p* = 0.000), denoting the presence of a considerably strong association between ED psychopathology and marital status. In line with our findings, obesity in patients with EDs is reported to be common among individuals who have only primary education—mostly patients with BN and BED (57).

In the whole sample and patients with other EDs, age, and education were significant predictors of depression while marital status was a marginal predictor. History of hospitalization predicted depression in the whole sample, but this effect vanished in either ED groups. Consistent with our findings, Anker et al. reported lower prevalence of depression and addictive disorders in ED patients with education above high school level (>12 years) (58). Women with chronic AN (disease chronicity >7 years) tend to be more depressed than short-term patients; they also are older and have more previous hospital admissions (55).

In this study, BMI predicted depression in the whole sample, but this effect was not noticed in either ED groups. Overeating usually occurs in response to negative emotions, and it contributes to obesity in some EDs such as BED (5). BMI correlates with suicidality (commonly associated with depression) in male and female patients with EDs, and this effect is aggravated by binging behavior (59). Pleplé et al. have shown an association between indicators of nutritional status (BMI and FFMI) and psychopathology (anxiety and depression) in hospitalized patients with AN. However, that association was confounded by psychotropic medications (antidepressant, anxiolytic, and neuroleptic drugs). These drugs were associated with lower FFMI at discharge, and FFMI was significantly associated with the severity of anxiety and depressive symptoms in these patients (2). Lower lifetime BMI in chronic patients with AN is associated with greater levels of depression and neuroticism than short-term patients (55). Therefore, the association between nutritional status and depression in EDs may be better investigated with regard to emotional dys/regulation, psychiatric treatment, and disease chronicity, which were all missing in the current study.

BMI predicted internet/Facebook addiction in all the samples, although its effect was marginal in both ED groups (Table 4). Consistent with our results, BMI is reported to be associated with internet addiction among adolescents, independent of ED subtype, age, and sex (60–62). However, one study reported no association between age, sex, and BMI with internet addiction among adolescents (27). The association between BMI and internet addiction may be explained by physical inactivity associated with prolonged internet use (>3 h) (60, 63, 64). Frequent snacking during use time may also affect BMI in individuals with internet addiction (60). This observation may hold true given the high prevalence of disordered eating attitudes evaluated by the Eating Attitude Test-26 (61) and disordered eating evaluated by the Eating Disorder Inventory among adolescents and college students with problematic internet use (20). In fact, prolonged computer use is associated with increased eating while not hungry, which promotes the build-up of excess body fat mass (64).

Young age predicted internet/Facebook addiction in all the samples, which is in accordance with several studies (17, 20, 65–67). Age seems to be a key factor in EDs. Among men and women, age demonstrates inverse linear associations with both restrictive and bulimic EDs, highlighting 18–25 years as the most vulnerable period (68). Age is also an important variable that correlates with several personality traits and behaviors. Young age is associated with lower educational level, which predicted internet/Facebook addiction in patients with other EDs. This finding is consistent with a former study reporting education as a confounding factor in neuropsychological alterations in individuals with BED (69). In our study, young age (commonly associated with being unmarried) negatively correlated with marital status (*r* = −0.524, *p* < 0.001), which marginally predicted depression and internet addiction in the whole sample. In harmony with this finding, university students who did not fall in love or failed their love relations were more likely to be Facebook dependent than students in love relations (66). Likewise, young Polish single women and women with higher scores on the De Jong Gierveld Loneliness Scale are reported to display the highest level of depressive symptoms and dependence on the internet and Facebook than older and married women (17). Likewise, internet addiction has a significant negative correlation with social support among university students (28). Altogether, young age, which entails high vulnerability to EDs, is associated with less education and being single/lonely, which may increase vulnerability to internet addiction.

In the current study, having a diagnosis of depression had no contribution to BMI, smoking, or problematic use of the internet and Facebook. In line with our findings, a cross-lagged panel study reported a moderate positive association between depression and internet addiction at different time points. However, in cross-lagged analysis, this association disappeared giving room to loneliness—which develops over time as a drawback of prolonged internet use—as a major predictor of internet addiction (63). In that study, online contact with family and friends could not prevent the development of loneliness associated with internet addiction (63). Facebook addiction is reported to be significantly higher in psychiatric inpatients with mild depression than in those with moderate depression (70). On the contrary, depression is reported to positively associate with Facebook addiction (66) and to partially mediate the relationship between internet addiction and weight change, weight concern, and binge eating among university students (20). Depressive and anxiety symptoms, and suicidal ideation are linked to excessive use of the internet in another sample of university students (25). Moreover, the literature shows that having a major depressive episode increases the risk for smoking and continued smoking in pregnant women (71). Likewise, analysis adjusted for age and gender shows that emotional dysregulation in smokers aggravates emotional eating resulting in increased BMI (5). The observed lack of associations between depression and other outcome variables in the present study may be attributed to the use of depression as a diagnosis rather than a symptom. Depression is a self-remitting disease—acute major depression regresses to the mean and improves spontaneously (72). The contribution of symptoms of depression or anxiety to dysfunctional behaviors goes far beyond having a diagnosed disease of depression or anxiety (16, 73). Cumulative knowledge associates symptoms of emotional negativity (anhedonia, negative affect, and low positive affect) with increased smoking risk, craving, and relapse although they demonstrate mixed or no relationship with smoking heaviness, chronicity, and nicotine dependence (74). These symptoms are not stable in people diagnosed with depression due to spontaneous recovery (72), while emotional symptoms of depression can alter psychosocial functioning (75). Longitudinal data show that depression severity was a key predictor of internet addiction among university students, especially younger students (65). In adjusted analysis, spontaneous remission of problematic internet use in adolescents was only predicted by lower levels of maladaptive emotion regulation strategies (76). Specific psychopathological features, including those occurring in depression (anhedonia and alexithymia) and those embroiled in emotional dysregulation such as dissociative proneness, are key correlates of binge drinking, problematic gambling, internet use, and physical exercise addiction among Italian adolescents (26). Therefore, absence of assessment of the severity and affective dimensions of depression as well as aspects of emotional dysregulation that contribute to depression in the current study disqualified the contribution of depression to BMI, tobacco smoking, and internet/Facebook addiction in the current study.

In our analysis, higher BMI, independent of depression, was a significant predictor of smoking only among patients with AN. This result indicates that smoking might be adopted as a weight control measure in this group of patients, which is in accordance with the available literature (35, 37). Observational studies show that higher BMI and body dissatisfaction in adolescent females are associated with increased odds of early onset regular smoking (36). Mendelian randomization analyses based on data from UK Biobank and the TAG consortium denote a causal effect of higher BMI on lifetime smoking, initiation of smoking, smoking heaviness, and DNA methylation at the aryl-hydrocarbon receptor repressor locus, but it has no effect on smoking cessation (34, 37). Smoking cessation plans were numerically higher in normal BMI Korean smokers than in underweight, overweight, or obese age- and gender-matched counterparts, although the difference was not significant (35). Other factors may affect smoking cessation such as high-risk drinking, levels of physical activity, and health problems [e.g., hypertension; (35)]. The contribution of these factors to smoking in patients with EDs is worthy of investigation in future studies.

In our study, smoking tended to be higher among older patients with all EDs and AN. In line, a meta-analysis reports that smoking rates are higher among adult, pregnant, and diseased women than in adolescent girls (40). In accordance, projection studies based on Spanish National Health Surveys (2003, 2006, and 2011) predict a significant decline in smoking rate among young Spanish women and a significant increase in smoking rate among older women (44). The level of education predicted smoking only in women with AN. This finding is consistent with the literature, which associates low educational levels with higher levels of smoking in EDs (68), other psychiatric conditions (77, 78), and even in healthy individuals (71).

Binge/purging subtype of AN as well as EDs characterized by excessive eating express genetic variability in dopamine D4 receptor (DRD4) (79, 80). DRD4 regulates reward response in different brain regions (e.g., prefrontal cortex) in drug addiction, food craving, and overeating. DRD4 mutations involving the 7R allele are associated with heavy smoking, higher craving, less dopamine release in the ventral caudate after smoking, less likelihood to smoking cessation, and greater reactivity to smoking-related cues in ex-smokers (79). It seems that genetic variability in DRD4 accounts for increased tendency for smoking among EDs, especially AN. In fact, nicotine vaping for 30 days among American university students (*N* = 51,231) is associated with increased self-reported lifetime EDs (all subtypes, 3.7%) and elevated ED risk (25.0%) (81). Another investigation of EDs among community-dwelling women with comorbid alcohol use disorder and nicotine dependence (*N* = 3,756, median age = 22 years) revealed that AN is the most prevalent disorder among these women (relative risk ratio, RRR = 3.17; 99% CI: 1.35–7.44) followed by purging disorder (RRR = 2.59; 99% CI: 1.24–5.43) and various symptoms of EDs (82). A similar investigation among individuals diagnosed with substance use disorder revealed a higher prevalence of food addiction than in the general population, especially among young women with high BMI (83). Although there was no significant association with any specific substance, the highest prevalence noted, in order, was among users of cannabis (31.03%), heroin (21.07%), cocaine (18.53%), alcohol (14.49%), and tobacco (11.61%) (83). Subchronic administration of the natural CB1/CB2 receptor agonist Δ9-tetrahydrocannabinol, found in cannabis, to rat models of activity-based AN can significantly reduce plasma corticosterone, weight loss, restrictive eating behavior, and running wheel activity (84). Involvement of the cannabinoid system in AN may also account for the high comorbidity of addictive disorders among AN patients, probably as an attempt for self-medication. Nonetheless, the present study did not properly assess the type of smoking (e.g., cigarette smoking vs. vaping) neither the intake of cannabis and other illicit drugs—a limitation in need to be addressed in future studies.

Chronicity was a negative predictor of smoking while being in treatment for a long time positively predicted smoking, especially in women with other EDs (Table 5). ED patients in prolonged treatment are likely to have a severe disease course with higher emotional eating, which may be associated with higher smoking (5). This logic is consistent with the fact that women with BN and binge/purge report the highest rates of smoking among all the subtypes of EDs (32, 33). ED patients receive antidepressant, anti-anxiety, and psychotropic drugs, which alter body composition and mood (2), in addition to modulating the cholinergic–nicotinic and dopaminergic systems (78). Therefore, smoking may be related to the neurobiological interactions induced by psychotropic medications while in prolonged treatment. In line with this argument, smoking and the use of hypnotic medications are frequently reported in individuals with nocturnal eating behavior and sleep-related eating disorder–like behavior, which express features consistent with bulimic and binge EDs (85). Likewise, adjusted analysis strongly relates smoking to the intake of psychotropic drugs in patients with schizophrenia (78). On the other hand, smoking may contribute to prolonged disease course because it causes interindividual variations in response to drug treatment. This is because the polycyclic aromatic hydrocarbons present in cigarette smoke induce hepatic aryl hydrocarbon hydroxylases resulting in higher metabolic clearance of substrate drugs for these enzymes (e.g., psychotropic and antidepressant drugs). As a result, biotransformation rates and plasma concentrations of these drugs decrease (86). This hypothesis may hold true given the cross-sectional nature of the current study, and it may be worth investigating in future studies.

In this study, smoking was not associated with internet addiction or Facebook addiction (*r* = 0.000 and −0.016, *p* > 0.05)—these paths were insignificant, and thus were trimmed in ML and Bayesian models to improve model fit. This finding is consistent with those of a study reporting marginal associations between Facebook/internet addiction and smoking or substance abuse in university students in Bangladesh (66). However, this result is contradictory to other studies reporting associations between smoking and internet addiction (26, 27, 29). It is not clear why the findings on the associations between smoking and internet addiction are mixed. However, this difference may be considered within the frame of desire frequency and strength for smoking reported by Hofmann et al. (87). Although smoking is addictive, they found that the averages of desire frequency and strength for smoking among adults are much lower and less conflicting than those for the desire for social media use. The latter were more likely to be enacted despite resistance (87). Nonetheless, studies are needed to investigate such a possibility.

This study has investigated several aspects of pathology in EDs: nutritional status, depression, internet addiction, Facebook addiction, and smoking. However, the generalizability of the findings of the current study may be limited by the cross-sectional design, inclusion of females only, single-clinical setting, unpowered and purposive sample, less representation of different subtypes of EDs, absence of assessment of negative affect, and possible social desirability bias. Internet/Facebook addictions were self-reported instead of being evaluated based on standard criteria of behavioral addiction described in common disease classification systems. It was not possible to evaluate many other key relations because of absence of various smoking-related variables (e.g., frequency, duration, vaping vs. cigarette smoking, age of initiation, use of smoking as a weight control strategy, taking part in smoking cessation programs, number of quitting trials, and relapse history). Moreover, relevant clinical characteristics of the sample were missing such as how the diagnosis of depression was established, presence of medical comorbidities and other addictive behaviors (e.g., alcohol and substance use), family history of psychiatric disorders, details of treatments (medications and psychotherapy) received by the participants, body dissatisfaction, and level of physical activity. In addition, absence of assessment of the key outcomes in age-cross-matched healthy control women in the present study may cast doubt on the possibility that the findings are specific to women with eating disorders. Replicating the study in larger samples, which comprise various subtypes of EDs from both genders, along with a healthy control group may be necessary to define the exact interaction among the addressed variables.

## Conclusion

This study highlights the contribution of age to BMI, depression, internet/Facebook addiction, and smoking in women with EDs. BMI contributed to internet/Facebook addiction and smoking in certain ED groups, but depression did not contribute to any of these variables. The level of education and history of hospitalization or prolonged treatment were associated with internet/Facebook addiction and smoking in certain ED groups. Given the devastating effects of co-morbid addictive behaviors on the disease course in EDs, early identification of addictive behaviors in highly prone ED patients becomes of specific importance. Empirical evidence documents positive effects of cognitive behavioral therapy (CBT) on internet addiction, anxiety, impulsivity, and social avoidance (88). For these conditions, CBT in combination with electro-acupuncture is more effective than CBT alone. Together, they increased the amplitude distance S1P50 and S2P50 of Auditory Evoked Potential, denoting that these interventions improve internet addiction symptom by increasing cerebrum sense perception gating function (89). Our findings pinpoint that interventional strategies targeting addictive behaviors in women with EDs may lead to promising results in young age, high BMI, single, and less educated groups.

## Data Availability Statement

The datasets presented in this study can be found in online repositories. The names of the repository/repositories and accession number(s) can be found at: Mendeley (https://data.mendeley.com/datasets/d5z2bnnv65/1).

## Ethics Statement

Ethical approval was not provided for this study on human participants because the analysis is based on a publicly available dataset. We are referring to the original study and dataset in our manuscript. Written informed consent to participate in this study was provided by the participants' legal guardian/next of kin.

## Author Contributions

AMA and HK conceptualized the topic. YK and HH acquired fund. AMA and HH conducted the statistical analysis and wrote the first draft of the article. HK and YK reviewed and edited the article. All authors have approved the final version of the article.

## Funding

This study was partially supported by the National Center Cohort Collaborative for Advancing Population Health funded by the Japan Health Research Promotion Bureau (JH) Research Fund [Project Number 2019-(1)-1].

## Conflict of Interest

The authors declare that the research was conducted in the absence of any commercial or financial relationships that could be construed as a potential conflict of interest.

## Publisher's Note

All claims expressed in this article are solely those of the authors and do not necessarily represent those of their affiliated organizations, or those of the publisher, the editors and the reviewers. Any product that may be evaluated in this article, or claim that may be made by its manufacturer, is not guaranteed or endorsed by the publisher.
